# Estimation of the Basic Reproductive Number and Mean Serial Interval of a Novel Pathogen in a Small, Well-Observed Discrete Population

**DOI:** 10.1371/journal.pone.0148061

**Published:** 2016-02-05

**Authors:** Kendra M. Wu, Steven Riley

**Affiliations:** 1 School of Public Health, LKS Faculty of Medicine, The University of Hong Kong, Hong Kong SAR; 2 Medical Research Council Centre for Outbreak Analysis and Modelling, Department of Infectious Disease Epidemiology, Imperial College London, London, United Kingdom; Harvard School of Public Health, UNITED STATES

## Abstract

**Background:**

Accurately assessing the transmissibility and serial interval of a novel human pathogen is public health priority so that the timing and required strength of interventions may be determined. Recent theoretical work has focused on making best use of data from the initial exponential phase of growth of incidence in large populations.

**Methods:**

We measured generational transmissibility by the basic reproductive number *R*_0_ and the serial interval by its mean *T*_*g*_. First, we constructed a simulation algorithm for case data arising from a small population of known size with *R*_0_ and *T*_*g*_ also known. We then developed an inferential model for the likelihood of these case data as a function of *R*_0_ and *T*_*g*_. The model was designed to capture a) any signal of the serial interval distribution in the initial stochastic phase b) the growth rate of the exponential phase and c) the unique combination of *R*_0_ and *T*_*g*_ that generates a specific shape of peak incidence when the susceptible portion of a small population is depleted.

**Findings:**

Extensive repeat simulation and parameter estimation revealed no bias in univariate estimates of either *R*_0_ and *T*_*g*_. We were also able to simultaneously estimate both *R*_0_ and *T*_*g*_. However, accurate final estimates could be obtained only much later in the outbreak. In particular, estimates of *T*_*g*_ were considerably less accurate in the bivariate case until the peak of incidence had passed.

**Conclusions:**

The basic reproductive number and mean serial interval can be estimated simultaneously in real time during an outbreak of an emerging pathogen. Repeated application of these methods to small scale outbreaks at the start of an epidemic would permit accurate estimates of key parameters.

## Introduction

Uncertainty dominates important early policy decisions for emerging respiratory pathogens such as Severe Acute Respiratory Syndrome coronavirus (SARS-CoV), Middle East respiratory syndrome coronavirus (MERS-CoV), and pandemic influenza A/H1N1. This uncertainty is reduced greatly once the basic reproductive number and the serial interval of infection are known. The basic reproductive number quantifies the transmissibility of a pathogen and is defined as the average number of secondary infections generated by one typically infectious individual in an otherwise susceptible population. The serial interval in each infector-infectee pair is the time between disease onset in the infector and the onset in the infectee.

In this study, we present a temporal likelihood model that allows real-time simultaneous estimation of *R*_0_ and the average of serial interval *T*_*g*_ in a small well-observed population, so that it provides timely information for informed public health responses. We build on previous inferential studies [1–4] by focusing on capturing unbiased parameter estimates as the epidemic leaves the exponential phase. Our two illustrative scenarios were designed to have similar exponential growth phases and are based loosely on pandemic influenza A/H1N1 and SARS-CoV infections.

## Methods

We first define the underlying stochastic process that simulates an epidemic, before describing our calculation of the likelihood of data arising from this simulated process and the parameter estimation based on this likelihood. The required inputs of the likelihood estimation are; the number of infected people from outside the community who triggered the epidemic, the size of susceptible in the community, and the case incidence time-series. Conversely, the primary outcomes are estimates of the basic reproductive number, *R*_0_, and the mean serial interval, *T*_*g*_.

### Simulate epidemic process

In the analysis, we defined our simulation process to produce the random time series

$$\begin{array}{c} X=\{X_{1},\ldots ,X_{j},\ldots ,X_{T}\} \end{array}$$

of new cases with onsets at time 1 to T. Specific realisations of **X** were defined to be **x** = {*x*_1_, …, *x*_*j*_, …, *x*_*T*_}.

We defined serial interval by assuming the probability that an individual with symptom onset on day *i* generates a new infection on day *i* + *τ* is

$$\begin{array}{c} f_{s}\left(\tau ,Tg\right)\propto \frac{Tg^{\tau }exp\left(-Tg\right)}{\tau !} \end{array}$$ (1)

where the right-hand side is the probability mass *τ* for a Poisson distribution with mean *T*_*g*_. Implicitly, this assumption about the serial interval implies that infectiousness was not constant for individuals in the days following their infection. The value of *k*, where *k* ≫ *T*_*g*_, was always sufficiently high that the probability of a secondary case was negligible after *k* relative to the infector’s onset time *i*. The offspring distribution [5] also assumed to be Poisson distributed with mean *R*_0_.

To initiate the simulation we assumed that there were *n* initially infectious individuals in a population of size *N*. Those initially infectious were assumed to have onsets at time *t*_1_. The simulation algorithm kept track of the onsets of the total number of infected individuals at each time unit **t** = {*t*_1_, …, *t*_*j*_, …, *t*_*T*_} during the observed period. Symbolically, the total number of infected individuals having onsets prior to and at time *j* was denoted as *I*_*j*_, where $I_{j}=\sum_{t=1}^{j}X_{t}$. For example *I*_1_ = *n*.

For an arbitrary infector of onset time *i*, we first simulated the number of secondary cases it had infected from the offspring distribution of mean *R*_0_, *f*_*O*_(*R*_0_), without considering the depletion of the susceptible population. For each of these secondary cases, we then drew the delay from the onset of the infector *i* to the onset of each secondary case from the serial interval distribution. The onsets fall between {*i* + 1, …, *i* + *k*} with mean *i* + *T*_*g*_. This process yielded the secondary case time-series **x** for a specific infector that had an onset time *i*.

When the number of infectees becomes significant relative to the susceptible population size, saturation effects must be accounted for. The effects are two-fold [6, 7]: first, through susceptible depletion, the number of secondary cases per primary case is reduced until there are no longer any susceptible people in the homogeneous population, which is when the number of secondary cases per primary case is reduced. Second, when there are many infectious cases, competition to infect the remaining susceptible shortens the serial interval. In this manuscript, we took the serial interval distribution to mean the distribution in the absence of competing sources of infection. The mean *T*_*g*_ was the average over of this distribution in the absence of competition.

In order to represent the finite pool of susceptible individuals, we defined the actual number of new cases with onsets at time *j* that accounted for the depletion of the susceptible population as $\tilde{z_{j}}$, and they were drawn from the as-yet uninfected pool using a binomial distribution, with probability of infection equal to the ratio of *z*_*j*_ and N:

$$\begin{array}{c} \tilde{z_{j}}∼B\left(N-I_{j-1},\frac{z_{j}}{N}\right). \end{array}$$ (2)

We repeated this process for each infector at time *i* and added up the secondary cases between {*i* + 1, …, *i* + *k*}. It should be noted that each infector produced different numbers of secondary cases and each of these infector-infectee pairs had different serial intervals. Each of these secondary cases were assumed to be infectious and were considered subsequent infectors if *i* > *j*. We repeated this process for all infectors with onset time between 1 and *T*. The overall case time-series **X** was the sum of each of the realization of secondary case time-series **x** of each infector.

### Likelihood

We defined *Y*_*j*_ to be the expected number of secondary cases of infectors *X*_*i*_ from {*j* − *k*, …, *j* − 1} with onsets at time *j* that could be expressed as,

$$\begin{array}{c} Y_{j}=\sum_{i=j-k}^{j-1}Y_{i}R_{0}f_{S}\left(j-i,T_{g}\right). \end{array}$$ (3)

where *f*_*s*_ was the Poisson density of serial interval.

We note that the formulation used above may not be correct for offspring and serial interval distributions that are not Poisson.

Therefore, the likelihood of a whole times series of observations could then be expressed in terms of the expected numbers of secondary cases in each time unit and the parameters of the model, when accounting to the finite pool of susceptible, as such:

$$\begin{array}{c} L\left(\tilde{Y}\right)=\prod_{j=2}^{T}p_{B}\left(\tilde{Y_{j}}|N-I_{j-1},\frac{Y_{j}}{N}\right) \end{array}$$ (4)

where $p_{B}$ was the probability mass function of a binomial distribution, and *Y*_*j*_ is defined in Eq (3).

Standard maximum likelihood were used to obtain point estimates and confidence intervals. We used the optim function in R [8] to find point estimates and the optimise function to find univariate confidence regions.

To initiate an epidemic and ensure that it would form a chain of transmission to establish an exponential phase of growth without early extinction, we decided to seed fifty infectious individuals on day 1 in each of our simulated epidemics. The subsequent number of incidences were then generated by the stochastic process described in the Simulate epidemic process.

All results in the paper can be reproduced using R code available in the github repository c97sr/EpiInference.

### Illustrative parameter regimes

We constructed two illustrative scenarios to investigate the properties of our model and likelihood function. Scenario 1: *R*_0_ = 1.8, *T*_*g*_ = 2.5 when using Poisson-distributed serial interval distribution; and Scenario 2: *R*_0_ = 3.0, *T*_*g*_ = 6.25 when using Poisson-distributed serial interval distribution. We chose these scenarios because they cover the range of SARS in hospitals (*R*_0_ = 2.7 [9]), pandemic A/H1N1 influenza in the community (*R*_0_ = 1.5 [10, 11]) and pandemic A/H1N1 in schools (*R*_0_ = 2.4 [12]). Then we compute *T*_*g*_ so that they had growth rate during the exponential phase was 0.32 according to $r=\frac{R_{0}-1}{T_{g}}$ [13]. Unless stated otherwise, we seeded day 1 with fifty individuals to avoid the epidemic prematurely died out. In each scenario, 1,000 people were assumed to be living in this community, where all but fifty were assumed to be susceptible initially. This population size was chosen to be consistent with a small town or large village in many social settings.

When using Poisson-distributed serial interval, we set *k* = 10 in Scenario 1 to simulate influenza infections [14] and *k* = 25 in Scenario 2 to loosely mimic SARS infections [15, 16]. They were chosen so that these values were at least four times greater than *T*_*g*_ and the probability of a secondary case after *k*, relative to the onset time *i* of the infector, was negligible. The effect of the truncation of the distribution was subsequently pushed to the right-hand tail of the estimate time-series when (*i* + *k*) > *T*.

## Results

**Fig 1:**
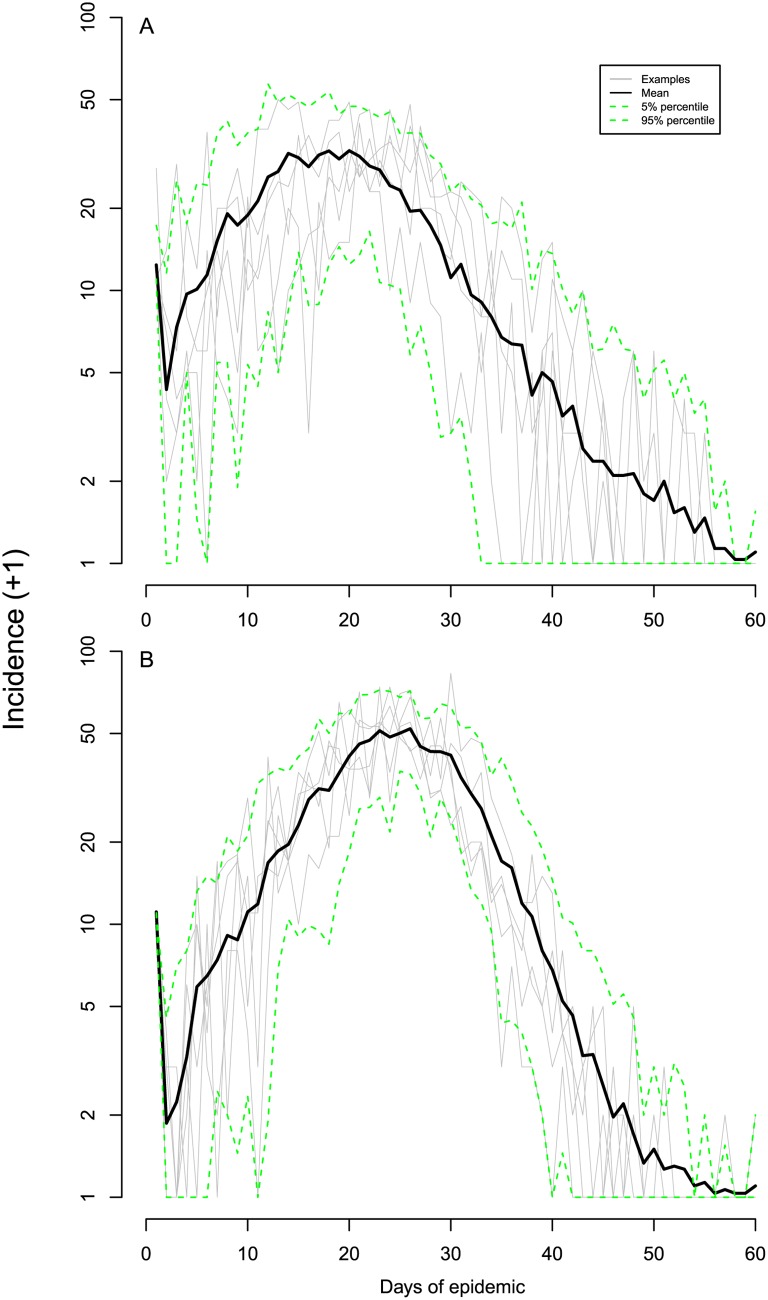
Simulated epidemics. Part **A** shows Scenario 1 with *R*_0_ = 1.8, *T*_*g*_ = 2.5. Part **B** shows Scenario 2 with *R*_0_ = 3.0, *T*_*g*_ = 6.25. Offspring and the serial interval assumed to follow Poisson distribution. In both scenarios, 1,000 individuals were initially susceptible and 10 people were infectious on day 1. Grey lines trace 5 single example realisations. Black lines show average case incidence, and the green dotted lines are the 5% and 95% prediction intervals from 30 realisations.

The different stages of an epidemic pass quickly in an outbreak of respiratory infections in a small population (Fig 1). In our simulated population of 1,000 people for an influenza-like pathogen (Scenario 1) there was an average delay of only 16.5 days (median, 95% prediction interval: 8.4, 26.2) from the introduction of 10 infectious individuals to peak incidence. The more transmissible, but slower Scenario 2 still achieved a peak of incidence after 23 days (18.1, 29.2). In addition to an early peak of incidence, partial saturation occurred very quickly in this population. An initial exponential increase in incidence can be seen on the log y-axes in Fig 1 (as a straight line). However, this exponential phase ends quickly in such a small population. In Scenario 1, incidence appears to be occurring sub-exponentially as early as 10 days after the initial seed.

**Fig 2:**
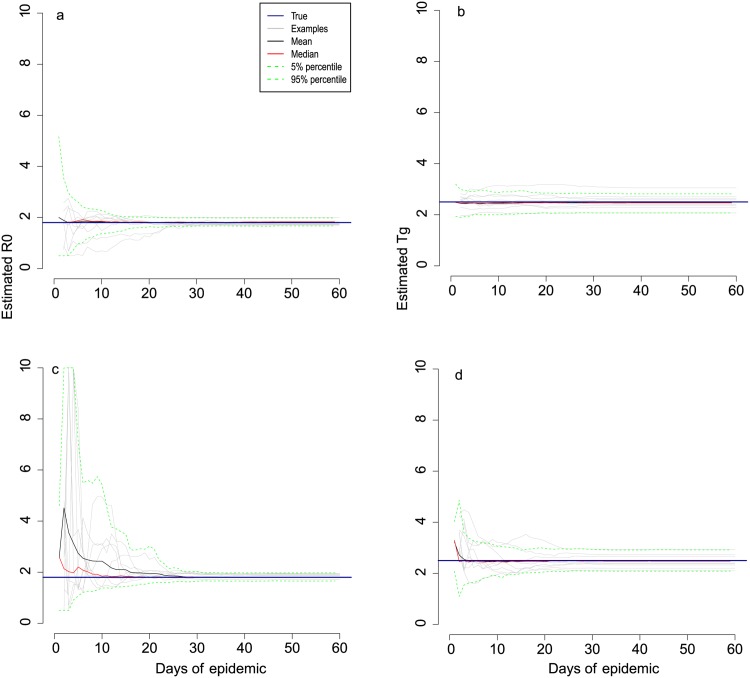
Univariate and bivariate parameter estimation from simulated daily data for Scenario 1. Part **a** shows real-time univariate estimates of *R*_0_ in which the true value of *T*_*g*_ was assumed known. Part **b** shows real-time univariate estimates of *T*_*g*_ with *R*_0_ assumed known. The results of jointly estimating *R*_0_ and *T*_*g*_ are shown in parts **c** (*R*_0_) and **d** (*T*_*g*_). Scenario 1 is as per Fig 1A with *R*_0_ = 1.8, *T*_*g*_ = 2.5. The epidemic was simulated in a population of 1,000 people all of whom were initially susceptible, other than 50 who were infectious on day 1. Grey lines trace the corresponding estimates of 8 arbitrary epidemics. Black lines represent the average of 100 estimates at that time point, red lines represent the median, and the green dotted lines are the 5% and 95% prediction intervals for 100 epidemics. The dark blue solid horizontal lines show the true parameter values.

**Fig 3:**
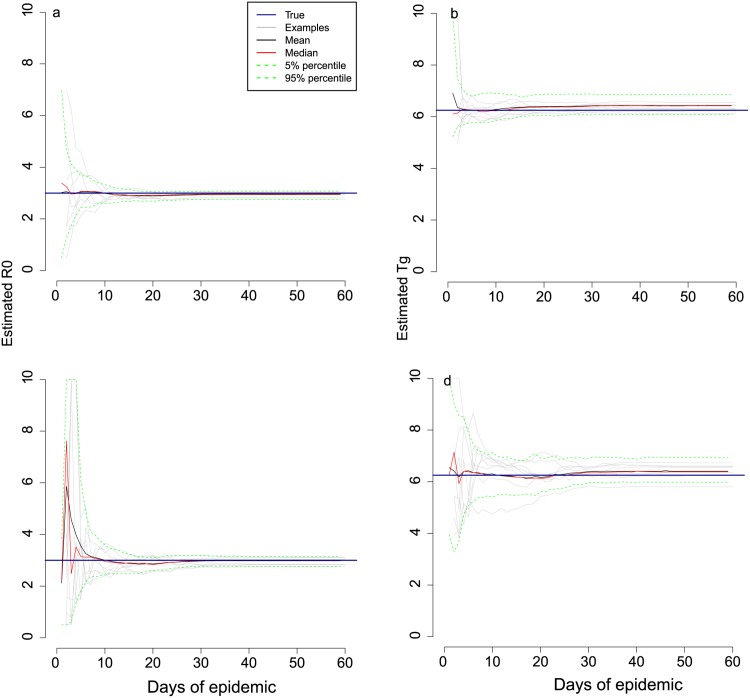
Univariate and bivariate parameter estimation from simulated daily data for Scenario 2. Part **a** shows real-time univariate estimates of *R*_0_ in which the true value of *T*_*g*_ was assumed known. Part **b** shows real-time univariate estimates of *T*_*g*_ with *R*_0_ assumed known. The results of jointly estimating *R*_0_ and *T*_*g*_ are shown in parts **c** (*R*_0_) and **d** (*T*_*g*_). Scenario 2 is as per Fig 1B with *R*_0_ = 3.0, *T*_*g*_ = 6.25. The epidemic was simulated in a population of 1,000 people all of whom were initially susceptible, other than 100 who were infectious on day 1. Grey lines trace the corresponding estimates of 8 arbitrary epidemics. Black lines represent the average of 100 estimates at that time point, red lines represent the median, and the green dotted lines are the 5% and 95% prediction intervals for 100 epidemics. The dark blue solid horizontal lines show the true parameter values.

The speedy transition from exponential growth to partial depletion of susceptible individuals and then peak incidence permitted rapid and accurate univariate parameter estimation. We attempted to recover either *R*_0_ or *T*_*g*_ (but not both at this stage) from simulated data similar to those described above. As would be expected, for both Scenario 1 (Fig 2a and 2b showed 8 arbitrary epidemics and prediction intervals of 100 epidemics) and Scenario 2 (Fig 3a and 3b showed 8 arbitrary epidemics and prediction intervals of 100 epidemics), we were able to obtain accurate values of *R*_0_ prior to the peak of the epidemics when *T*_*g*_ was known, even during the earliest exponential phase of incidence. Estimates of *R*_0_ did not become any more accurate once the peak of incidence had passed. Although the pattern of univariate inference for *T*_*g*_ was similar to that for *R*_0_, the final confidence intervals were wider (in relative terms) than for *R*_0_.

**Fig 4:**
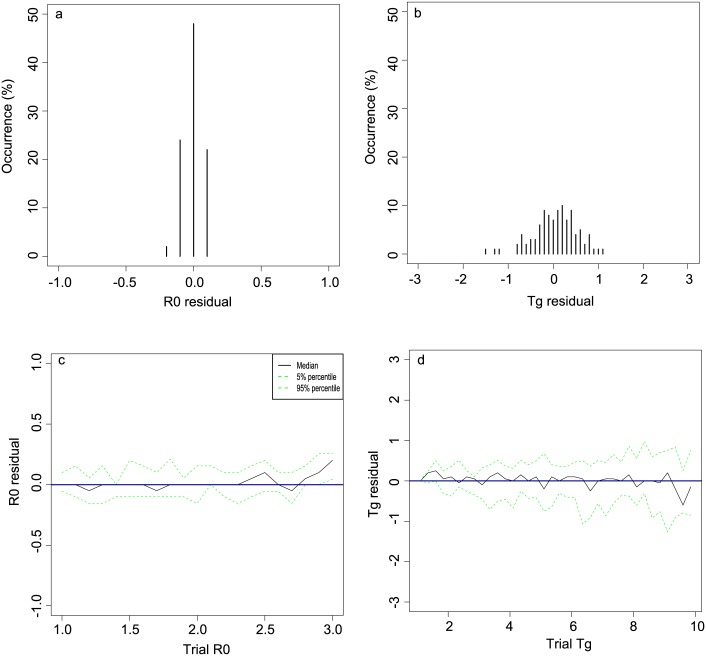
Extended assessment of univariate inference for Scenario 1. Sensitivity analysis using univariate estimations at single time point and assumed poission-distributed serial interval. **a** and **b** show the distribution of the marginals for estimation of *R*_0_ and *T*_*g*_ of 50 realisations respectively. All estimates were made at day 50. **c** shows the mean and confidence intervals for the marginals for estimates for *R*_0_ across a wide range of known values for *T*_*g*_. Similarly, **d** shows the mean and confidence intervals for *T*_*g*_ across a wide range of value for *R*_0_. Scenario 1 is as per Fig 1A with *R*_0_ = 1.8, *T*_*g*_ = 2.5, and *k* = 10.

We extended our analysis of the univariate parameter estimation routines to test the model’s performance (Fig 4 for Scenario 1). For a day 50 in the simulated epidemic of 50 realizations, we held *R*_0_ and *T*_*g*_ constant in the simulated epidemics while varying the values of a) *R*_0_ estimates, denoted as $\hat{R_{0}}$, or b) *T*_*g*_ estimates, denoted as $\hat{T_{g}}$, by ±1.5 in the likelihood function. Next, we repeated the process by c) varying *R*_0_ from 1.0 to 3.0 and d) varying *T*_*g*_ from 1.1 to 10.0 in the simulated epidemics, then computed the residuals of c) $\hat{R_{0}}$ and d) $\hat{T_{g}}$ from the likelihood function respectively as previously done.

Nevertheless, it was possible to jointly estimate both *R*_0_ and *T*_*g*_ in this small population. We assumed that neither *T*_*g*_ nor *R*_0_ were known for an emerging pathogen and attempted to estimate them jointly for Scenario 1 (Fig 2c and 2d showed 8 arbitrary epidemics and prediction intervals of 100 epidemics) and Scenario 2 (Fig 3c and 3d showed 8 arbitrary epidemics and prediction intervals of 100 epidemics). Compared to the univariate cases, for both scenarios, reliable information was obtained on both *R*_0_ and *T*_*g*_. However, prediction intervals were wider. Once the peak had been reached, the convergence of estimates of *T*_*g*_ in bivariate case were achieved, but in relative terms, the accuracy was considerably lower.

The estimates of different values of *k* were tabulated on Table 1 for the model using Poisson-distributed serial interval for the estimates on the last day of observation using different numbers of iterations: (i) 10, (ii) 20, (iii) 100, along with different *k* values.

**Table 1:** Univariate and bivariate estimates of the model using Poisson distributed serial interval, different *k* values, and number of simulation iterations. The following parameters were used: (a) *R*_0_ = 1.8, *T*_*g*_ = 2.5, *k* = 10; (b) *R*_0_ = 1.8, *T*_*g*_ = 2.5, *k* = 25; (c) *R*_0_ = 3.0, *T*_*g*_ = 6.25, *k* = 10; (d) *R*_0_ = 3.0, *T*_*g*_ = 6.25, *k* = 25. PI: prediction interval.

		**Univariate estimations**					
**Parameters**	**Iterations**	**R0 estimates**			**Tg estimates**		
		**Mean**	**Median**	**(5%, 95%)**	**Mean**	**Median**	**(5%, 95%)**
a	10	1.74	1.74	(1.58, 1.90)	2.55	2.50	(2.15, 2.97)
	20	1.79	1.81	(1.66, 1.90)	2.50	2.44	(2.06, 2.91)
	100	1.79	1.79	(1.63, 1.93)	2.48	2.48	(2.01, 2.96)
b	10	1.80	1.77	(1.73, 1.90)	2.50	2.53	(2.12, 2.79)
	20	1.81	1.80	(1.66, 2.00)	2.48	2.41	(2.17, 3.04)
	100	1.82	1.81	(1.70, 1.96)	2.46	2.47	(2.07, 2.82)
c	10	2.99	3.01	(2.76, 3.16)	6.27	6.22	(6.06, 6.65)
	20	2.99	3.02	(2.82, 3.14)	6.31	6.28	(5.89, 6.63)
	100	2.99	2.99	(2.82, 3.18)	6.31	6.31	(5.95, 6.68)
d	10	2.93	2.94	(2.75, 3.06)	6.33	6.38	(6.01, 6.57)
	20	2.92	2.91	(2.80, 3.07)	6.40	6.45	(6.02, 6.68)
	100	2.99	2.99	(2.77, 3.22)	6.28	6.24	(5.91, 6.72)
		**Bivariate estimations**					
		**Mean**	**Median**	**(5%, 95%)**	**Mean**	**Median**	**(5%, 95%)**
a	10	1.74	1.73	(1.58, 1.91)	2.50	2.41	(2.18, 2.89)
	20	1.78	1.80	(1.64, 1.89)	2.48	2.47	(2.01, 2.97)
	100	1.79	1.78	(1.64, 1.91)	2.47	2.46	(1.99, 2.98)
b	10	1.80	1.80	(1.71, 1.94)	2.50	2.47	(2.09, 2.90)
	20	1.81	1.79	(1.69, 1.98)	2.48	2.48	(2.19, 2.92)
	100	1.81	1.81	(1.71, 1.95)	2.48	2.48	(2.09, 2.92)
c	10	2.99	3.00	(2.75, 3.18)	6.26	6.26	(5.97, 6.64)
	20	3.01	2.99	(2.79, 3.18)	6.32	6.32	(6.04, 6.68)
	100	3.01	2.99	(2.81, 3.24)	6.32	6.29	(5.99, 6.78)
d	10	2.92	2.94	(2.71, 3.09)	6.23	6.18	(5.89, 6.61)
	20	2.96	2.92	(2.75, 3.18)	6.31	6.31	(5.80, 6.74)
	100	3.00	2.97	(2.77, 3.27)	6.27	6.24	(5.88, 6.70)

## Discussion

We have described a quantitative method that allows the rapid joint estimation of the basic reproductive number, and mean and variance of serial interval for an emerging pathogen based only on case data from an outbreak in a small population. These estimates show no signs of bias and could be available quickly for small populations. In essence, this approach is able to tease apart the two parameters from case data because it extracts key information from the transmission dynamics of different phases of a respiratory epidemic: the initial stochastic period of growth, the deterministic period of exponential growth, the sub-exponential period of growth prior to peak incidence, and the peak itself. Previous similar work [1, 2, 4] has incorporated either the initial stochastic phase or one or more of the deterministic phases, but not all phases together.

The serial interval distribution plays a crucial role in the estimation accuracy. It is because when the variance was large, the estimator had more difficulty to tease apart the two parameters from the observed incidence data, especially during phase II and beyond. In this model, the serial intervals are assumed to follow a Poisson distribution. When it has large variance and thus less distinguishable between generations of infection comparing to a shape Gamma distribution, the joint estimations have poorer performance, although they remain reasonably accurate in infinite population [3]. In finite population, the estimations of $\hat{T_{g}}$ showed no bias (Table 1) if it had sufficiently large seed size.

By seeding fifty and a hundred infectious people on day 1 in each epidemic, the number of incidences at the early stage of the outbreaks would likely be higher and plausibly reached the exponential phase of growth earlier than if there were smaller number of infectious individuals seeded. However, with the same transmissibility and speed of transmission, higher number of incidences and large seed size appear to have no impact to the accuracy of estimates aside from avoiding outbreaks prematurely died out.

Our study is intended as an initial theoretical illustration of the potential for this approach to be applied more widely. As such, it suffers from a number of limitations caused directly by the restrictive assumptions we have made. For example, we have assumed that all cases in this small population were observed. However, despite the possibility of intense surveillance in a small population, it is unlikely that even all symptomatic cases will be detected. Also, most pathogens generate some mild or entirely asymptomatic cases that would evade even the most intense surveillance efforts. Although we doubt, based on related previous study [17] that the reporting of only a proportion of cases would bias results (if that proportion is constant), it would most likely increase the delay from the start of the epidemic to the acquisition of robust parameter estimates.

We have also assumed throughout that offspring distribution and serial interval distribution follow a Poisson density. These are convenient assumptions that allow very rapid likelihood calculations for it is a single-parameter defining function. We anticipate few theoretical issues in the extension of these approaches to include distributions with more than one parameter (other than careful modification of Eq (3) as indicated above). However, the ability of these methods to independently estimate both means and variances of the offspring distribution and serial interval distribution remains to be described. We would anticipate that the estimation of variance for the serial interval distribution would be challenging [18] for cases other than where there are very high volumes of data for the first few generations (which could arise from a large seeding event) or when the true distribution has a low variance.

There are likely substantial opportunities to gain valuable insights into outbreak dynamics if we relax our assumption that transmissibility is constant over time and thus consider an inferential framework that accounts for both changes in transmissibility and depletion of susceptible individuals. The reproduction number would vary over time because of both effects. This approach might be especially useful for severe infections that affect small populations, such as Ebola.
